# Comparing Depressive Symptoms in the Health and Retirement Study and Mexican Health and Aging Study

**DOI:** 10.1093/geroni/igaf122.058

**Published:** 2025-12-31

**Authors:** Nicholas Bishop

**Affiliations:** University of Arizona, Tucson, Arizona, United States

## Abstract

Cross-national examination of depressive symptoms and comorbid conditions can help identify mechanisms that can be leveraged to reduce the burden of depression among Mexican-origin older adults, but the comparability of depressive symptom indicators in the Health and Retirement Study (HRS) and Mexican Health and Aging Study (MHAS) is currently untested. I used the 2018 HRS (n = 1,267) and MHAS (n = 10,883), 7 CESD-based depressive symptom indicators that were asked in both studies (felt depressed, alone, sad, happy, everything an effort, restless sleep, enjoy life), and self-reported somatic chronic conditions (hypertension, diabetes, cancer, stroke, arthritis) to assess measurement invariance across depressive symptom indicators, then examined how chronic conditions were associated with depressive symptom factor scores. The largest difference in standardized factor loadings existed for enjoy life (Δ = 0.17) and felt happy (Δ = 0.13), but all loadings were sufficient to assume configural invariance. Approximate fit indices (e.g., comparative fit index [CFI] and McDonald’s non-centrality fit index [MFI]) were used to assess measurement invariance. Across assessments of weak, strong, and strict factorial invariance, changes in CFI and MFI were negligible (max CFI Δ = 0.001, max MFI Δ = 0.004), suggesting strict invariance could be assumed. When predicting depressive symptom factor scores in multiple-group models adjusted for age, sex, education, and number of doctor visits in past year, arthritis demonstrated the strongest association with depressive symptoms, followed by heart attack, hypertension, and diabetes. These associations did not significantly differ across studies. These preliminary results suggest select depressive symptom indicators in the HRS and MHAS can be used to make cross-national comparisons.

